# Mutation analysis of *aryl hydrocarbon receptor interacting protein* (*AIP*) gene in colorectal, breast, and prostate cancers

**DOI:** 10.1038/sj.bjc.6603573

**Published:** 2007-01-23

**Authors:** M Georgitsi, A Karhu, R Winqvist, T Visakorpi, K Waltering, P Vahteristo, V Launonen, L A Aaltonen

**Affiliations:** 1 Department of Medical Genetics, Molecular and Cancer Biology Research Program, University of Helsinki, 00014 Helsinki, Finland; 2 Departments of Clinical Genetics and Oncology, Oulu University Hospital/University of Oulu, Oulu OYS 90029, Finland; 3 Institute of Medical Technology and Tampere University Hospital, University of Tampere, 33014 Tampere, Finland

**Keywords:** pituitary adenoma, PAP, *AIP*, colorectal tumours, breast tumours, prostate tumours

## Abstract

Germline mutations in the *aryl hydrocarbon receptor interacting protein* (*AIP*) gene were recently identified in individuals with pituitary adenoma predisposition (PAP). These patients have prolactin (PRL) or growth hormone (GH) oversecreting pituitary adenomas, the latter exhibiting acromegaly or gigantism. Loss-of-heterozygosity (LOH) analysis revealed that *AIP* is lost in PAP tumours, suggesting that it acts as a tumour-suppressor gene. Aryl hydrocarbon receptor interacting protein is involved in several pathways, but it is best characterised as a cytoplasmic partner of the aryl hydrocarbon receptor (AHR). To examine the possible role of *AIP* in the genesis of common cancers, we performed somatic mutation screening in a series of 373 colorectal cancers (CRCs), 82 breast cancers, and 44 prostate tumour samples. A missense R16H (47G>A) change was identified in two CRC samples, as well as in the respective normal tissues, but was absent in 209 healthy controls. The remaining findings were silent, previously unreported, changes of the coding, non-coding, or untranslated regions of *AIP*. These results suggest that somatic *AIP* mutations are not common in CRC, breast, and prostate cancers.

Germline mutations of *aryl hydrocarbon receptor interacting protein* (*AIP*), a gene coding for a cytoplasmic interaction partner of aryl hydrocarbon receptor (AHR) (also known as dioxin receptor), were recently described to cause low-penetrance pituitary adenoma predisposition (PAP) (Vierimaa *et al*, 2006).

Pituitary adenomas are common benign neoplasms of the anterior pituitary gland and account for approximately 15% of intracranial tumours. About two-thirds oversecrete pituitary hormones, among which growth hormone (GH) and prolactin (PRL)-oversecreting adenomas are the most common (Heaney and Melmed, 2004). The causal effect of GH oversecretion is acromegaly or gigantism. Cardiovascular, cerebrovascular, respiratory, and metabolic diseases are potential more severe consequences (Melmed *et al*, 1995; Colao *et al*, 2004; Jenkins, 2004).

There is accumulating evidence for an increased risk of colorectal cancer (CRC) in acromegaly patients, but also circumstantial evidence that breast and prostate malignancies may arise at a higher risk in the context of acromegaly (Jenkins, 2004). An attractive explanation for the increased risk of CRC in acromegaly has been the link to insulin-like growth factor I (IGF-I). Plasma GH triggers the production of IGF-I from the liver, which in turn stimulates the growth of organs and tissues, through its known mitogenic and antiapoptotic properties. Any imbalance in the tight control between epithelial cell turn-over and cell death may result in epithelial hyperproliferation, promoting the formation of hyperplastic polyps and colorectal adenomas (Jenkins *et al*, 2006). Chan *et al* (1998) have reported a strong positive association between IGF-I levels and increased prostatic malignancy , whereas Peyrat *et al* (1993) have described increased IGF-I levels in primary breast cancers.

We recently described the PAP phenotype in pituitary adenoma patients from Northern Finland (Vierimaa *et al*, 2006). Germline *AIP* mutations segregated with the PAP phenotype in the familial cases, and germline mutations were also identified in sporadic acromegaly patients from Northern Finland. Loss-of-heterozygosity (LOH) analysis revealed loss of wild-type allele in all tumour samples available, suggesting that *AIP* is likely to act as a tumour suppressor gene.

Aryl hydrocarbon receptor interacting protein is best characterised through its cytoplasmic interaction with the AHR (Carver and Bradfield, 1997), a transcription factor that regulates many xenobiotic metabolising enzymes (Meyer and Perdew, 1999; Marlowe and Puga, 2005). Aryl hydrocarbon receptor interacting protein modulates the stability and subcellular localisation of AHR by preventing its dynamic nucleocytoplasmic shuttling (Pollenz and Dougherty, 2005). Aryl hydrocarbon receptor interacting protein has also been reported to interact with phospho diesterase 4A5 (PDE4A5) (Bolger *et al*, 2003) and peroxisome proliferator-activated receptor-α (PPAR-α) (Sumanasekera *et al*, 2003).

Many genes involved in hereditary cancer syndromes are also somatically mutated in tumours that are not prominently associated with the respective syndromes (Li *et al*, 1997; Avizienyte *et al*, 1999; Ali *et al*, 1999; Sanchez-Cespedes *et al*, 2002). The identification of *AIP* as a candidate tumour-suppressor gene, lost in both GH and PRL-secreting pituitary adenomas, prompted us to study its possible involvement in other tumour types. According to the global cancer statistics reviewed by Parkin *et al* for the year 2002, breast (18%), colorectal (12%), and prostate cancers (10%) are, in terms of prevalence, the most common worldwide (Parkin *et al*, 2005). To characterise the possible contribution of somatic *AIP* mutations to the genesis of common cancers, we performed a mutation analysis in a series of CRC, breast, and prostate cancer samples. The inclusion of breast cancer samples was also based on a recent report from Eltom *et al* (2006), that the levels of nuclear AHR – the cytoplasmic interaction partner of AIP – were dramatically elevated in human breast carcinomas of advanced malignancy compared to earlier stages of tumour progression. In the tumours of advanced malignancy, the nuclear AHR was stabilised and constitutively active.

## MATERIALS AND METHODS

### Sample material

The study was approved by the appropriate ethics review committees. Deoxyribonucleic acid from 52 CRC samples (44 microsatellite stable (MSS) and eight microsatellite unstable (MSI), chosen from a series collected between 1994–1998; Aaltonen *et al*, 1998; Salovaara *et al*, 2000) were used for *AIP* mutation analysis. *Aryl hydrocarbon receptor interacting protein* exon 1 screening was subsequently extended to 321 CRC samples (272 MSS and 49 MSI) also chosen from the same series collected between 1994 and 1998. The whole set of 373 CRC patients had a mean age at diagnosis of 68 years (range 30–88 years). Colorectal cancer samples were collected as fresh-frozen tissue specimens and displayed at least 50% tumour tissue, according to pathologist's histological evaluation. Corresponding normal tissue DNA was extracted from blood or normal colonic epithelium, distant from the tumour margins.

A total of 82 breast cancer DNA samples were available for the study. The samples were derived regardless of family history, from patients diagnosed between 1988 and 1994 at the Oulu University Hospital (Winqvist *et al*, 1995). Complete *AIP* screening was initially performed in 43 samples. Subsequently, *AIP* exon 6 screening was extended to 39 additional tumour samples. The 82 individuals had a mean age at diagnosis of 54 years (range 29–87 years).

In addition, 44 unselected, genome Phi-amplified (GenomiPhi Amplification kit, Amersham, GE Healthcare, UK), prostate tumour samples from the Tampere region (Waltering *et al*, 2006) were analysed. Of these, 28 were previously untreated cancers (primary), 12 were hormone-refractory, and four were characterised as benign prostate hyperplasia. The individuals had a mean age at diagnosis of 66 years (range 56–87 years). DNA from 209 anonymous, cancer-free Finnish Red Cross blood donors served as a population-matched control material.

### Mutation screening

The coding region, exon–intron boundaries, as well as the 5′ and 3′ untranslated regions (UTRs) of *AIP* were PCR-amplified and sequenced as previously described by Vierimaa *et al* (2006). The potential splicing effect of an intronic change in one breast tumour sample was predicted by computational methods using NetGene2 (http://www.cbs.dtu.dk/services/NetGene2) and the Berkley Drosophila Genome Project (BDGP) (http://www.fruitfly.org/seq_tools/splice.html) splice site prediction programs. The protein sequences of human AIP and its homologues in other species were obtained from the University of California in Santa Cruz (UCSC) Genome Bioinformatics database, version March 2006 (http://genome.ucsc.edu), and Ensembl Genome Browser, version 38–April 2006 (http://www.ensembl.org).

## RESULTS

**Figure 1:**
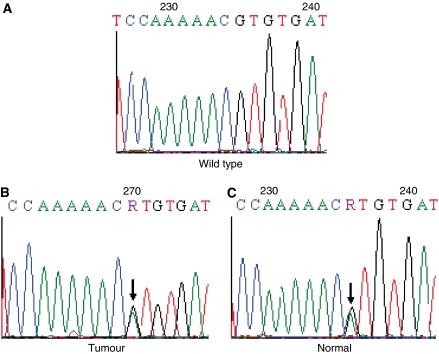
*Aryl hydrocarbon receptor interacting protein* germline change R16H found in two CRC samples. Here, the MSS sample is depicted as example. (**A**) Exon 1 wild-type sequence, shown for comparison. (**B**) R16H detected in the tumour sample. (**C**) R16H detected in the respective normal sample. Loss-of-heterozygosity was not observed in either of the cases with the R16H variant. The mutated base 47G>A is indicated by an arrow.

Among the 52 CRC samples initially screened, a heterozygous missense change, R16H (47G>A) in exon 1 was detected in two samples, one being MSS and the other MSI (Table 1). Sequencing of the corresponding normal tissues showed the presence of R16H in the germline in both cases (Figure 1). The change was absent in 209 healthy controls. The MSS patient had been diagnosed with cancer of the rectum at the age of 64 years. Owing to unilateral breast enlargement, the patient had been examined by nuclear magnetic resonance (NMR) for pituitary adenoma. However, owing to restricted access to medical records in the private sector, and given that this patient is now deceased, no detailed data were available. The MSI patient had been diagnosed with cancer of the colon at the age of 81 years. Her sister had been also diagnosed with CRC at the age of 78 years, and her daughter with cervical cancer at the age of 33 years. Interestingly, her brother had been diagnosed with carcinoid tumour of the rectum at the age of 66 years.

Not knowing whether R16H is a very rare polymorphism or a pathogenic mutation related to colorectal cancer, we extended the screening of exon 1 to an additional series of 321 CRC samples, but no additional R16H carriers were observed. In one of these samples, we found a heterozygous G>C change in the 5′ UTR, 5 bp upstream of the start codon (Table 1). This patient had been diagnosed with cancer of the rectum at the age of 72 years. The change was present in the patient's germline and absent in 209 healthy controls.

A second heterozygous missense change in exon 1, G23E (68G>A), was identified in six out of the total of 373 (0.8%) CRC samples. This change was also observed in five out of 209 healthy controls (1.2%) (Table 1). Finally, a previously unreported heterozygous G>C variant of the 3′ UTR, 60 bp downstream of the termination codon, was identified in two MSI samples out of the total of 373 CRC tumours (0.3%). The variant was also observed in three out of 182 (0.8%) healthy controls in heterozygous form.

The screening of breast tumour samples also revealed the G>C change in the 3′ UTR, 60 bp downstream of the termination codon. The change was present in six out of 81 (4.3%) breast tumours, of which five were heterozygotes and one was homozygote for the rare C allele (Table 1). The variant was also observed in three out of 182 (0.8%) healthy controls in heterozygous form (*P*=0.026, Fisher's exact test). In addition, one breast tumour sample had a heterozygous change at IVS3+15C>T (Table 1). This change was not predicted to have a splicing effect, as tested *in silico* by NetGene2 and BDGP programs.

In prostate tumour samples, the only observed *AIP* alterations were two previously unreported variants, likely to be silent polymorphisms, in one sample each (Table 1): G12G (36G>A) coding for glycine in exon 1 in a primary cancer sample and C238C (714C>T) coding for cystein in exon 5 in a hormone-refractory cancer sample. *AIP* mutation screening results are summarised in Table 1.

## DISCUSSION

Recently, we reported that *AIP* is a low-penetrance tumour susceptibility gene, conferring genetic predisposition to pituitary adenomas with high relative, but low absolute, risk (Vierimaa *et al*, 2006). Little is known concerning the involvement of AIP in pituitary tumorigenesis or progression, as its functional role in that context remains to be clarified. Here, we wished to examine whether *AIP* is somatically mutated in the most common cancer types: Colorectal, breast, and prostate cancer. No somatic *AIP* mutations were detected in this study, in accordance with a recent study by Sjöblom *et al* (2006); *AIP* was not found to be mutated in human breast and CRC cancer.

**Figure 2:**
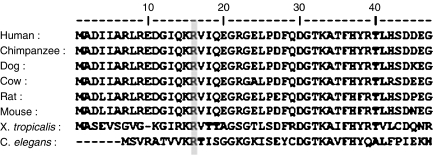
Alignment of human AIP amino-acid sequence and its homologues among species. The conserved arginine (coded by human *AIP* codon 16) is highlighted by a grey box.

The presence of the missense change R16H in the tumour and corresponding normal tissue (Figure 1) of two CRC patients is an interesting finding. First, the fact that arginine at *AIP* codon 16 is a highly conserved amino acid among species (Figure 2), second, from the possibility that an occult pituitary adenoma had been present in the MSS patient in which R16H was identified, one could speculate that such an occult PRL and GH-secreting adenoma may have promoted the breast enlargement, as well as the CRC. Third, the presence of carcinoid tumour in the brother of the MSI R16H-positive patient seems interesting; carcinoids are neuroendocrine tumours, which often occur as part of complex familial endocrine cancer syndromes, such as Multiple Endocrine Neoplasia Type 1 (Leotlela *et al*, 2003); however, no material was available for segregation studies of the R16H change in this family. *Aryl hydrocarbon receptor interacting protein* analyses in additional CRC cases and healthy controls would shed light on this possibility, and clarify whether or not R16H is associated with neoplasia. We have subsequently encountered the R16H variant in multiple other individuals with endocrine tumours, but also in one healthy control (unpublished observations). Thus, it is too early to conclude whether this change is pathogenic or not.

We next wanted to examine the possibility that *AIP* is somatically mutated in unselected breast cancers. Among the breast tumours analysed, we found no pathogenic changes, but only silent, previously unreported, polymorphisms. We presume that the presence of the 3′ UTR +60 bp G>C change in a subset of breast tumour samples, as well as in the two MSI CRC samples, is incidental. The individuals with the variation were somewhat, but not significantly, younger at diagnosis than those without the variation (46±12.57 *vs* 54.82±13.6 years; *P*⩽0.13, Student's *t*-test). The clinical data of the six individuals having the 3′ UTR+60 bp G>C change are not uniform: two were familial, one being *BRCA1* mutation-positive (4216 nt-2A>G), and the second being *BRCA1*/*2* mutation-negative. All six breast tumours were unilateral; five were infiltrating ductal carcinomas, whereas the familial *BRCA1*/*2* mutation-negative was of medullary type. The malignancy status was also heterogeneous: two were of advanced and two of intermediate malignancy, but no information was available for the remaining two samples. These breast cancer patients originate from the Oulu region of Northern Finland and the 3′ UTR+60 bp G>C change might represent a variation that is enriched in this population, as it was also observed in three healthy controls. We thus assume that the 3′ UTR+60 bp G>C change is a previously unreported polymorphism.

No pathogenic changes were identified in the series of prostate tumour samples; two silent variants in *AIP* exons 1 and 5, respectively, were detected and are likely to be polymorphisms. These findings suggest that *AIP* mutations are not common in breast and prostate cancers.

Despite the high success rate of *AIP* DNA amplification (99%) and the sensitivity of our sequencing analysis system, we might have failed to detect some changes that escape detection by conventional polymerase chain reaction (PCR) and direct genomic sequencing. Such changes might include large genomic rearrangements or promoter methylation, which are common mechanisms for tumour suppressor gene inactivation. On the other hand, our results are consistent with the fact that some genes mutated in certain hereditary tumour syndromes are rarely somatically mutated in sporadic tumours. For instance, *BRCA1*/*2* are the two major breast and ovarian cancer susceptibility genes. However, somatic mutations of *BRCA1/2* in sporadic breast tumours, as well as in other cancer types, are very rare (Futreal *et al*, 1994; Khoo *et al*, 1999; Naderi and Couch, 2002; Yang *et al*, 2002).

The mechanisms by which *AIP* participates in tumorigenic events in the pituitary are unknown. The present study does not provide strong evidence for immediate involvement of *AIP* in the initiation or progression of colorectal, breast, and prostate cancer. It remains to be examined whether *AIP* is somatically mutated in other tumour types more related to pituitary adenomas, such as various neuroendocrine tumours.

## Figures and Tables

**Table 1:** *AIP* changes identified in colorectal, breast, and prostate tumour samples

**Cancer type**	**Number of patients with a variant**	**Location**	**Nucleotide change**	**Amino-acid change**	**Individuals (allele frequencies)**	**Controls (allele frequencies)**
Colorectal	2	Exon 1	47G>A	R16H	2/373 (0.27%)	0/209
	6	Exon 1	68G>A	G23E	6/373 (0.8%)	5/209 (1.2%)
	1	5′ UTR	−5G>C	—	1/373 (0.13%)	0/209
	2	3′ UTR	+60G>C (heterozygous)	—	2/373 (0.27%)	3/182 (0.8%)
						
Breast	1	Intron 3	IVS3+15C>T	—	1/43 (1.16%)	NA
	5	3′ UTR	+60G>C (heterozygous)	—		
	1		+60G>C (homozygous)	—	6/81 (4.3%)	3/182 (0.8%)
						
Prostate	1	Exon 1	36G>A	G12G	1/44 (1.14%)	0/209
	1	Exon 5	714C>T	C238C	1/44 (1.14%)	NA

NA=not analysed; UTR=untranslated region.
